# Resolution of Paraneoplastic Cutaneous Angioendotheliomatosis After CAR T‐Cell Therapy in TP53‐Deleted CLL: A Case Report

**DOI:** 10.1002/jha2.70358

**Published:** 2026-07-30

**Authors:** Feras Alfraih, Mostafa F. Mohammed Saleh, David Li, Razan Mohty, Mohamed Kharfan‐Dabaja

**Affiliations:** ^1^ Adult Hematology, Stem Cell Transplant and Cellular Therapy Department Oncology Center King Faisal Specialist Hospital and Research Center Riyadh Saudi Arabia; ^2^ Pathology and Laboratory Medicine Mayo Clinic Jacksonville Florida USA; ^3^ Division of Hematology‐Oncology University of Alabama Birmingham Alabama USA; ^4^ Blood and Marrow Transplantation Program Division of Hematology‐Oncology Mayo Clinic Jacksonville Florida USA

**Keywords:** CAR T‐cell therapy, case report, chronic lymphocytic leukemia, cutaneous lesions, TP53 mutation

## Abstract

Cutaneous manifestations of chronic lymphocytic leukemia (CLL) are heterogeneous and may result from leukemic infiltration, secondary malignancies, infectious complications, or reactive inflammatory processes. Reactive angioendotheliomatosis (RAE) is a rare vascular proliferation that has only infrequently been reported in association with hematologic malignancies. We describe a 58‐year‐old man with high‐risk relapsed/refractory CLL harboring *TP53* mutation and del(17p) who developed rapidly enlarging, fungating cutaneous lesions involving the right thumb, upper arm, and additional sites despite multiple prior therapies, including fludarabine, cyclophosphamide, rituximab, ibrutinib, and venetoclax. Histopathologic evaluation demonstrated dermal vascular proliferation with endothelial marker positivity (CD31, CD34, ERG, and vimentin), consistent with RAE and without evidence of leukemic infiltration. The lesions were refractory to local supportive measures but showed marked regression following treatment with CD19‐directed chimeric antigen receptor (CAR) T‐cell therapy using lisocabtagene maraleucel. Notably, cutaneous remission despite persistent measurable residual disease and eventual systemic relapse requiring allogeneic hematopoietic cell transplantation. This discordance suggests that regression of RAE may be mediated through immune modulation and alteration of inflammatory or angiogenic pathways rather than direct elimination of cutaneous leukemia. To our knowledge, this is among the first reports describing resolution of paraneoplastic RAE following CAR T‐cell therapy in CLL, and it highlights the broader immunomodulatory effects of cellular immunotherapy beyond direct antitumor activity.

**Trial Registration:**

The authors have confirmed clinical trial registration is not needed for this submission

## 1 Introduction

Chronic lymphocytic leukemia (CLL) is the most common adult leukemia in Western countries, characterized by clonal proliferation of mature B lymphocytes. While the disease typically involves peripheral blood, bone marrow, and lymphoid tissues, extranodal and extramedullary infiltration can occur, most often in the skin, reported in fewer than 5% of patients. Cutaneous manifestations of CLL are diverse, encompassing specific leukemic infiltration (leukemia cutis), secondary malignancies, infectious complications, and inflammatory dermatoses [1].

Histopathology is required to distinguish leukemic infiltration from mimicking conditions such as infections, drug reactions, or secondary skin cancers. While leukemia cutis does not typically alter prognosis, it can be disfiguring, recurrent, and resistant to conventional therapies. The advent of targeted agents and cellular immunotherapies, including chimeric antigen receptor (CAR) T‐cell therapy, provides new therapeutic opportunities in this setting.

## 2 Case Description

**FIGURE 1:**
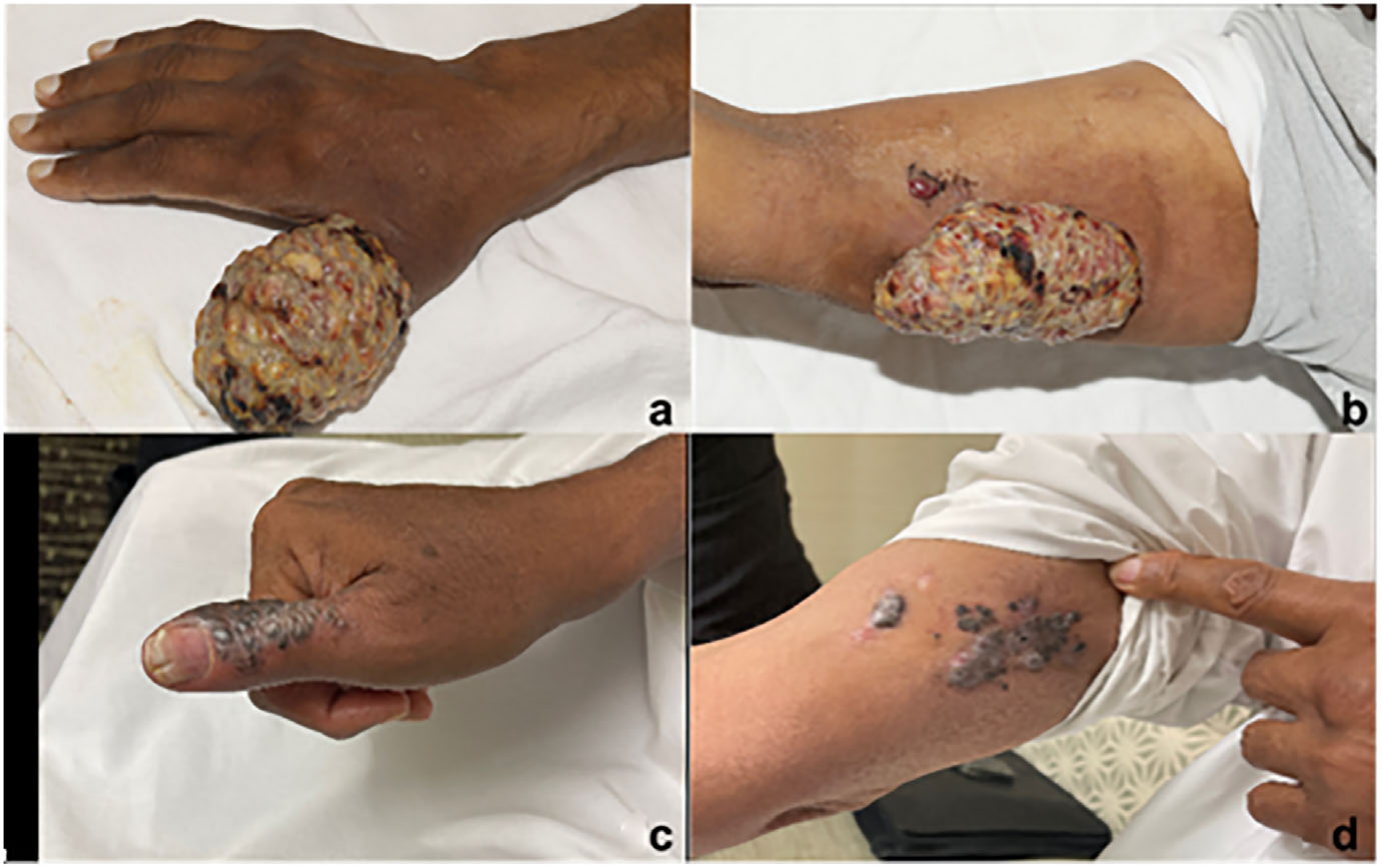
Skin lesions in a patient with relapsed/refractory CLL. (a, b) Lesions prior to CAR T‐cell therapy. (c, d) Lesions after CAR T‐cell therapy.

**FIGURE 2:**
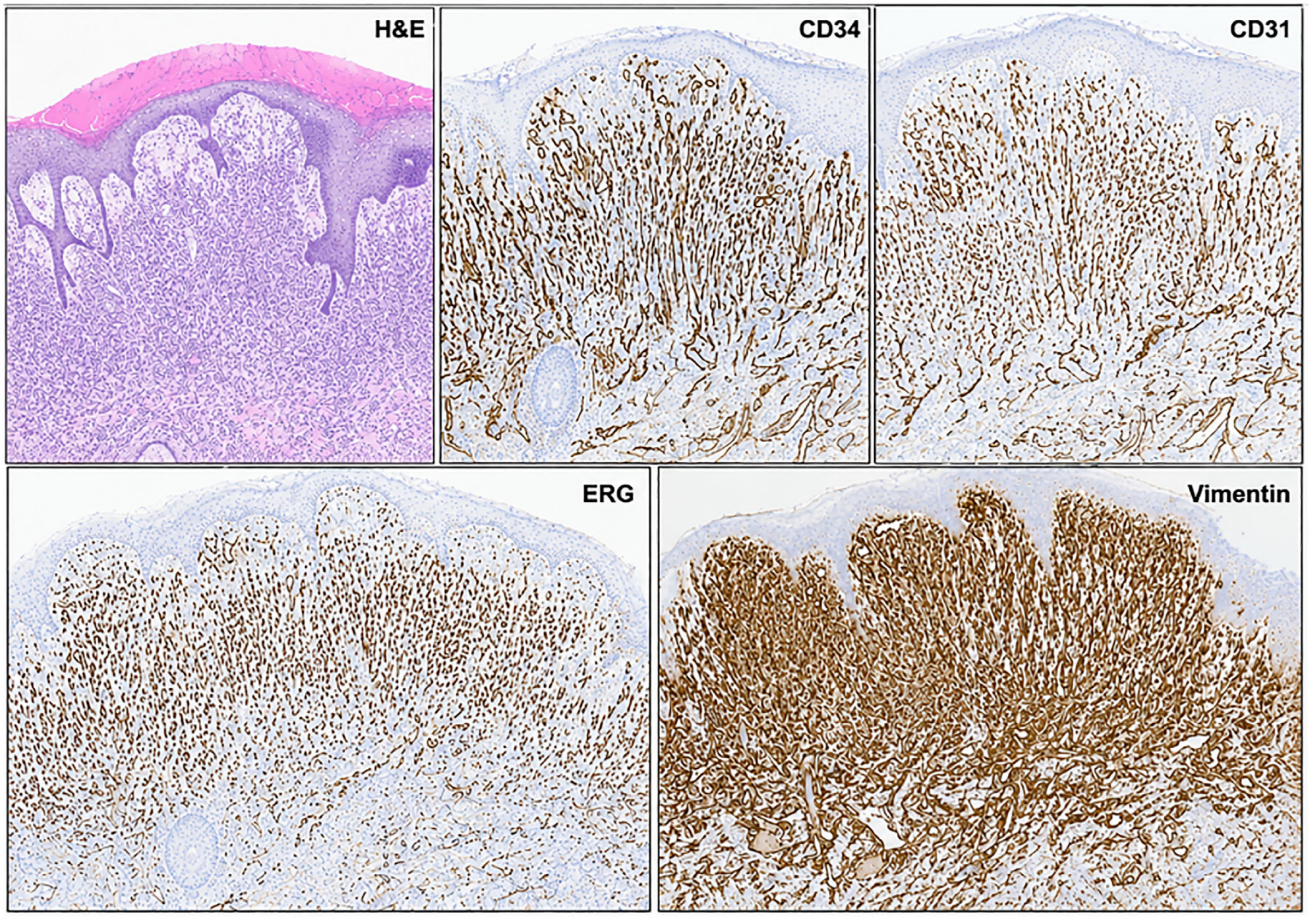
Histopathological and immunohistochemical features of the skin lesion. (a) Hematoxylin and eosin (H&E) staining demonstrates a polypoid dermal lesion with prominent vascular proliferation beneath an acanthotic epidermis (original magnification ×40). Immunohistochemistry shows diffuse positivity for (b) CD34, (c) CD31, (d) ERG, and (e) vimentin, supporting the vascular nature of the lesion (original magnification ×40).

A 58‐year‐old male with high‐risk relapsed/refractory CLL harboring *TP53* mutation and del(17p) presented for evaluation after receiving multiple lines of therapy, including chemoimmunotherapy with fludarabine, cyclophosphamide, and rituximab (FCR), ibrutinib; and venetoclax. His presentation also included a rapidly enlarging lesion over the right thumb (Figure 1a) and right anterior upper arm (Figure 1b). The lesions evolved from a painless nodule into a fungating, exophytic mass with superimposed infection. Initial excision and biopsy outside our institution revealed no malignancy, likely reflecting sampling limitations. However, the lesions recurred within weeks, accompanied by the development of a similar mass at the left forearm graft donor site and new subcutaneous nodules over the limbs and trunk. Dermatologic evaluation raised differential diagnoses, including giant pyogenic granuloma, pyogenic granuloma‐like reaction, and angiosarcoma. An additional punch biopsy of the right anterior upper arm showed reactive angioendotheliomatosis (RAE) by demonstrating dermal vascular proliferation with variably lobular architecture that was uniformly positive for CD31, CD34, ERG, and vimentin (Figure 2). The lesions were managed with antibiotics and wound care without noticeable improvement.

He received CD19‐directed CAR T‐cell therapy with lisocabtagene maraleucel in the context of a clinical trial. Following treatment, the skin lesions showed marked regression, with resolution of exophytic components and healing by secondary intention, leaving behind sclerotic, hyperpigmented plaques (Figure 1c,d). Despite improvement in involved skin areas, systemic disease persisted with detectable minimal residual disease, and subsequent clinical relapse necessitated an allogeneic hematopoietic cell transplant. At follow‐up, there was no reactivation of the skin lesions, and no new nodular or inflammatory cutaneous findings. The prior thumb lesion showed post‐inflammatory hyperpigmentation and mild fibrosis, while the forearm donor site exhibited flattened scarring. These cutaneous manifestations likely reflected a reactive paraneoplastic phenomenon temporally linked to systemic disease activity.

## 3 Discussion

Cutaneous manifestations in CLL are diverse and frequently atypical. In a retrospective review of more than 750 patients, Agnew et al. identified 40 patients with 125 skin lesions, including secondary cancers, viral infections, inflammatory dermatoses, and leukemia cutis, with many showing atypical clinical or histologic features independent of prior therapy [2]. Such variability complicates diagnosis and underscores the importance of histopathologic evaluation in suspected cases. In the present case, histopathological assessment confirmed RAE, characterized by benign intravascular endothelial proliferation without evidence of leukemic infiltration, supported by CD31, CD34, ERG, and vimentin positivity. This distinction is critical, as RAE represents a paraneoplastic vascular phenomenon rather than a manifestation of extramedullary CLL.

The risk of skin malignancies in CLL has important prognostic implications. In a large cohort, Velez et al. demonstrated that non‐basal cell skin cancers, particularly squamous cell carcinoma, were associated with mortality rates equivalent to CLL itself, with advanced Rai stage and higher tumor stage predicting poor outcomes [3]. These findings highlight the interplay between CLL biology and cutaneous disease, necessitating multidisciplinary management to optimize outcomes. Leukemia cutis itself remains rare but clinically relevant. Aldapt and Yassin's review of 56 cases showed head and neck predominance, frequent papulonodular lesions, and good overall response to treatment, with 78% achieving remission and no clear impact on prognosis [4]. Cerroni et al. previously described characteristic histologic patterns in 42 patients, noting that skin involvement could precede systemic diagnosis in ∼17% of cases, while Richter transformation carried a poor prognosis [5]. In addition, Lu et al. reported a patient with long‐standing CLL who developed widespread cutaneous infiltration involving the ears, eyebrows, nose, buttocks, fingers, and toes [6]. Remarkably, despite declining chemotherapy, the patient survived 3 years with only mild symptom progression, underscoring that even extensive skin disease does not uniformly predict poor survival but can present with highly atypical and disfiguring features. More recent reports highlight therapeutic advances. Robak et al. described accelerated CLL with extensive LC resistant to multiple regimens, achieving remission with venetoclax plus rituximab [7]. Similarly, Blasco et al. reported toe infiltration in an elderly woman with untreated CLL, successfully managed with chlorambucil and obinutuzumab [8]. These cases illustrate both the heterogeneity of presentation and the potential responsiveness of cutaneous disease to modern targeted therapies.

In contrast to leukemia cutis, the mechanism underlying the resolution of RAE following CD19‐directed CAR T‐cell therapy is likely indirect and multifactorial. Because RAE lacks a malignant CD19‐positive cellular target, regression cannot be attributed to direct cytotoxic activity within the skin. Instead, resolution likely reflects modulation of systemic disease‐driven inflammatory or angiogenic signaling, leading to normalization of endothelial proliferation. Furthermore, CAR T‐cell therapy induces a robust immune activation state characterized by cytokine release, including interleukin‐6 and interferon‐gamma [9], which may contribute to vascular remodeling and restoration of endothelial homeostasis.

An intriguing aspect of this case is the discordance between durable cutaneous remission and subsequent systemic relapse. Although the skin lesions were initially temporally associated with active disease, their sustained resolution despite measurable residual disease and eventual clinical progression suggests a degree of biological uncoupling between paraneoplastic manifestations and tumor burden. This observation raises the possibility that CAR T‐cell therapy induces durable immune or microenvironmental changes that persist beyond its antileukemic effect [10]. Potential mechanisms include prolonged immune effector activity, cytokine‐mediated suppression of endothelial proliferation, or localized tissue‐specific immune reprogramming that renders the cutaneous microenvironment less permissive to recurrence.

Previous literature has largely focused on leukemia cutis, with limited data addressing reactive vascular phenomena such as RAE. However, paraneoplastic vascular proliferations have been described in association with hematologic malignancies and are thought to arise from dysregulated cytokine and angiogenic pathways [11]. Our case expands this spectrum by demonstrating that cellular immunotherapy may effectively reverse such paraneoplastic processes, even in the absence of durable systemic remission.

Importantly, these findings do not support a “sanctuary‐site” effect in this context, as no leukemic infiltration was identified within the skin. Rather, they underscore the broader immunomodulatory capacity of CAR T‐cell therapy beyond direct tumor cytotoxicity. Further studies are needed to better define the interplay between immune effector therapies, paraneoplastic phenomena, and tissue‐specific responses in CLL.

## 4 Conclusion

RAE is a rare paraneoplastic cutaneous manifestation of CLL that may mimic leukemia cutis. This case demonstrates durable resolution of RAE following CD19‐directed CAR T‐cell therapy despite subsequent systemic relapse, suggesting that immune‐mediated mechanisms beyond direct antileukemic activity may contribute to cutaneous disease control.

## Author Contribution

All authors contributed equally to writing and editing of the manuscript and approved submission of the final version.

## Funding

The authors have nothing to report.

## Ethics Statement

This case report was reviewed and approved by the institutional review board of our institution. All procedures were conducted in accordance with institutional guidelines and the principles of the Declaration of Helsinki.

## Consent

Written informed consent was obtained from the patient for publication of this case report, including all clinical information and accompanying images.

## Conflicts of Interest

The authors declare no conflicts of interest.

## Data Availability

Data are available upon reasonable request.
